# Long-Term Pulmonary Damage From SARS-CoV-2 in an Infant With Brief Unexplained Resolved Events: A Case Report

**DOI:** 10.3389/fmed.2021.646837

**Published:** 2021-06-11

**Authors:** Luana Nosetti, Massimo Agosti, Massimo Franchini, Valentina Milan, Giorgio Piacentini, Marco Zaffanello

**Affiliations:** ^1^Lombardy Regional Sudden Infant Death Syndrome Center, Division of Pediatrics, F. Del Ponte Hospital, University of Insubria, Varese, Italy; ^2^Department of Neonatology, Neonatal Intensive Care Unit, and Pediatrics, F. Del Ponte Hospital, University of Insubria, Varese, Italy; ^3^Department of Hematology and Transfusion Medicine, Carlo Poma Hospital, Azienda Socio Sanitaria Territoriale, Mantova, Italy; ^4^Division of Pediatrics, F. Del Ponte Hospital, Varese, Italy; ^5^Department of Surgical Sciences, Dentistry, Gynecology, and Pediatrics, University of Verona, Verona, Italy

**Keywords:** infant, newborn, sleep disorders, breathing, COVID-19, pneumonia

## Abstract

A brief unexplained resolved event (BRUE) is an event observed in a child under 1 year of age in which the observer witnesses a sudden, brief but resolved episode of change in skin color, lack of breathing, weakness or poor responsiveness. Severe acute respiratory syndrome coronavirus 2 (SARS-CoV-2) is the causative agent of coronavirus disease-2019 (COVID-19). We report the case of a previously healthy, full-term infant infected with SARS-CoV-2 when he was 8 months old. Previous to this event, both his grandfather and great-uncle had died of severe pneumonia and his mother had developed respiratory symptoms and fever. Over the following month he was seen five times in the emergency room and was hospitalized twice for recurrent BRUE. At the first hospital admission, after the second emergency room visit, he twice tested positive for COVID-19 after nasopharyngeal swab tests. During his second hospital admission, after the fifth emergency room visit, chest computed tomography revealed typical SARS-CoV-2 pneumonia. During a follow-up examination 6 months later, mild respiratory distress required administration of inhaled oxygen (0.5 L/min) and chest computed tomography disclosed a slight improvement in pulmonary involvement. The clinical manifestation of pulmonary complications from COVID-19 infection was unusual. This is the first report of an infant at high-risk for BRUE, which was the only manifestation of long-term lung involvement due to SARS-CoV-2 pneumonia.

## Introduction

The severe acute respiratory syndrome coronavirus 2 (SARS-CoV-2) is a novel coronavirus first identified in Wuhan, China, and the etiological agent of coronavirus disease-2019 (COVID-19). It is spread by human-to-human transmission via droplets or direct contact. The estimated average incubation period is 6.4 days (1). Evidence suggests that, in addition to the direct cytopathic effect of SARS-CoV-2, there is also the indirect effect of cell-mediated immunity, cytokine storm, and cross-talk between organs resulting in possible multisystem failure (2). The most common sites of SARS-CoV-2 infection are the respiratory tract when the virus is inhaled. Lung damage severity is closely related to the severity of infection, which progresses with viral replication and the release of pro-inflammatory cytokines (3). The imaging features of SARS-CoV-2 include bilateral patchy ground-glass opacities, extensive bilateral interstitial and air space opacities (4).

A brief unexplained resolved event (BRUE) is an event observed in an infant younger than 1 year of age during which the observer witnesses a sudden, brief self-limiting episode of change in skin color, shortness of breath, weakness or poor reactivity. The event usually occurs suddenly, lasts between 30 and 60 s, and is frightening to the person caring for the infant (5). High-risk infants are younger than 2 months of age, have a history of pre-maturity and more than one previous event (6). Here we describe the case of a previously healthy infant in which the SARS-CoV-2 infection progressed to chronic pneumonia and presented as high-risk for BRUE.

## Case Report

A male infant was born at-term after an uncomplicated delivery. He had always been in good health before current presentation. His grandfather and great uncle had both died from severe pneumonia and his mother had developed respiratory symptoms and fever when he was 8 months old. His parents took him to the emergency department of another hospital in the north of Italy because of hyperpyrexia (maximum 39°C) that resolved 3 days later. He was treated at home with an anti-inflammatory drug. No blood chemistry tests or instrumental investigations were performed at that time.

Ten days later, his parents brought him to the emergency room again because of a 3-day history of dyspnea and abdominal breathing. Medical examination, chest X-ray, baseline electrocardiogram, and peripheral oxygen saturation (SpO_2_) were normal. Biochemistry tests showed a mild increase in serum creatine kinase (CK; 322 IU/L; reference range, 38–234 IU/L). The child was discharged in good health.

On his third emergency department room visit, he was hospitalized for 15 days due to reported infrequent brief episodes of apnea, which resolved spontaneously after a change in position. On hospital admission, blood chemistry tests showed mild metabolic acidosis, mild hypoglycemia (63 mg/dL), and CPK normalization (214 IU/L); a chest X-ray showed initial peri-bronchial consolidations and a nasopharyngeal swab tested positive for COVID-19 twice. The child was discharged in good health after the nasopharyngeal swab tested negative for COVID-19.

Shortly thereafter, he reportedly experienced bradypnea at home and difficulty waking up despite repeated painful stimuli from his parents. On admission to the emergency department, he appeared healthy. The pediatric neurology examination was normal and he was discharged.

**Figure 1:**
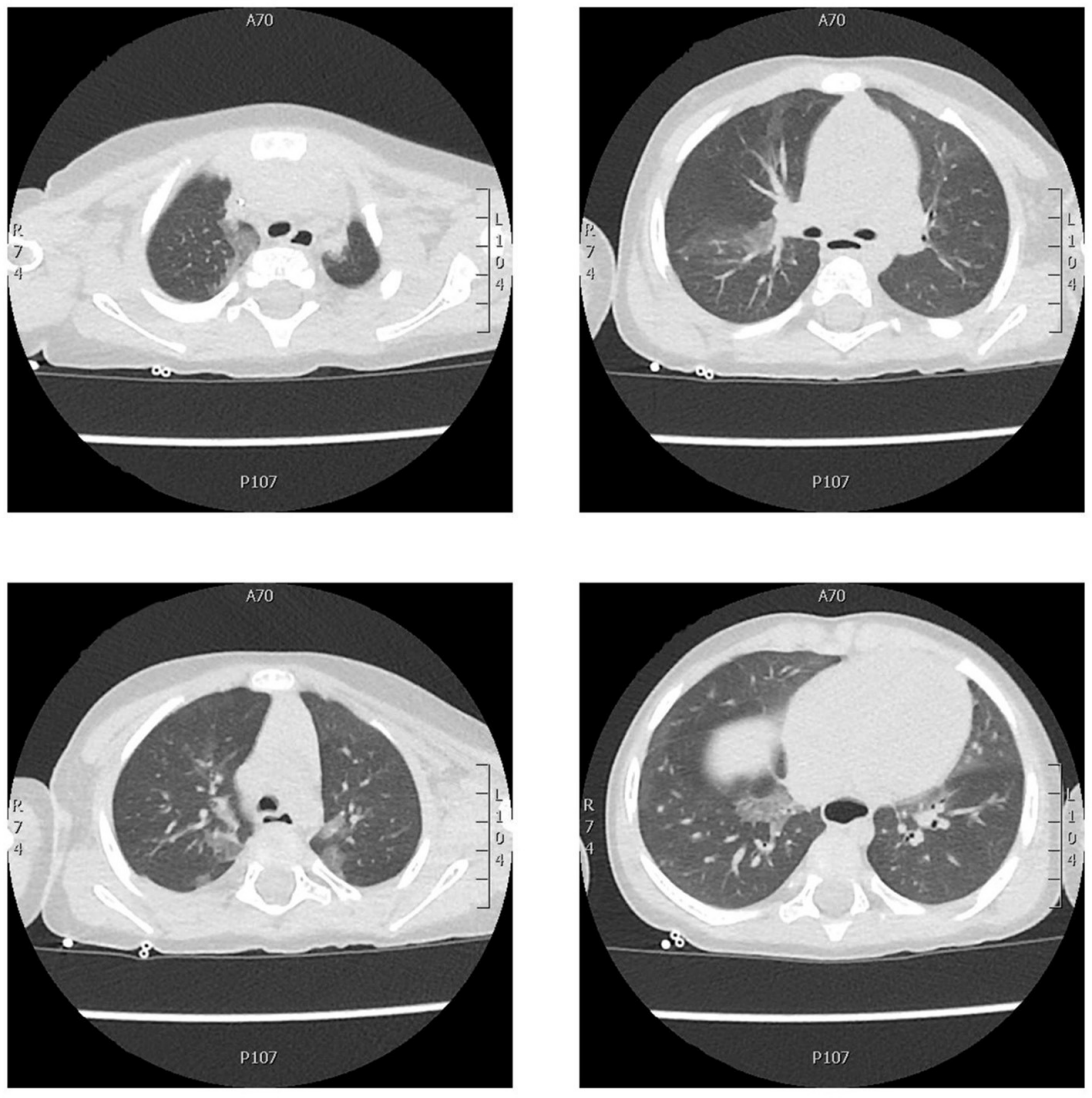
Chest CT axial scan showing areas of central and peripheral ground-glass opacity extending from the apex to the base of both lungs with no pleural effusion. The estimated total lung involvement was 5–10%.

Twelve days later, the parents noted apnea, hypotonia, and skin pallor and brought him to the emergency department. The medical examination was normal. Because of the unexplained and repeated emergency department visits, he was referred to the Lombardy Regional Center for SIDS (sudden infant death syndrome) and BRUE. On admission, the SpO_2_ was 91–95%, the body temperature 36.1°C, the respiratory rate 36 breaths per min, and the heart rate 120 beats per min. Chest auscultation revealed initial right basal hypophonesis and rales with small sub crepitating bubbles. Cell blood count, C-reactive protein (CRP), and blood chemistry were all normal. SARS-CoV-2 neutralizing antibodies were 247 AU/ml (positive >15 AU/ml). Findings of blood and diagnostic exams performed on second hospitalization is showed in the Table 1. The chest X-ray was normal. An electrocardiogram (ECG) QTc was 440 msec. The echocardiogram was normal with no pericardial effusion. Chest computed tomography (CT) showed areas of central and peripheral ground-glass opacity extending from the apex to the base of both lungs. The estimated total lung involvement was 5–10% (Figure 1). Full polysomnography recorded short episodes of apnea lasting <15 s, mean SpO_2_ 98%, and minimum SpO_2_ 94.5%. Transcutaneous oximetry (ptCO_2_) in ambient air was 47 mm Hg. He had difficulty breathing and received 0.5–1 L/min of oxygen administered by nasal cannula and oral steroids with benefit. He was discharged in good health and his parents were instructed to continue with home cardiorespiratory monitoring and to return for periodic outpatient visits.

**Table 1:** Findings of blood and diagnostic exams performed on hospital admission and follow-up.

**Characteristic**	**Second hospitalization**	**Third hospitalization (5–6 months later)**
WBCs (/mmc)	8,490	8,630
RBCs (/mmc)	4,740,000	4,210,000
Hb (gr/dl)	12.4	11.5
Platelet count (/mm^3^)	325,000	231,000
CRP (mg/L)	<0.3	<0.6
Serum creatinine (μmol/L)	18.5	22.1
ALT (U/l)	48	Normal
AST (U/l)	23	Normal
LDH (U/l)	NA	290
Creatin kinase (IU/l)	88	Normal
LDH (U/L)	NA	290
Nasopharyngeal swab SARS-CoV-2 RNA test	Positive	Negative
Serology	Influenza Virus A and B, and RSV negative	Adenovirus, Rhinovirus, Influenza Virus A, B negative, RSV negative
SARS-CoV-2 Neutralizing Antibodies (AU/ml)	247	114
Chest X-ray	Normal	Normal
Electrocardiogram and ultrasound	Normal	Normal
Chest computed tomography	Central and peripheral ground-glass opacity from the apex to the base of both lungs	Central and peripheral ground-glass opacity in the left para-mediastinal area, superior and inferior lobes
Full polysomnography	Apnea (<15 s)	NA
ptCO_2_	47 mm Hg	35 mm Hg
Neurological exam and EEG	Normal	NA
Medications	Steroids	Inhaled oxygen, salbutamol inhalant, steroids, azithromycin.

Five months after his second hospitalization, he received a regularly scheduled vaccination against measles/rubella/mumps and a pneumococcal vaccination 1 month later. His parents brought him again to the emergency department because of increased breathing and difficulty breathing after mild effort and hypoxia (transcutaneous SpO_2_ 88–89%) during sleep.

**Figure 2:**
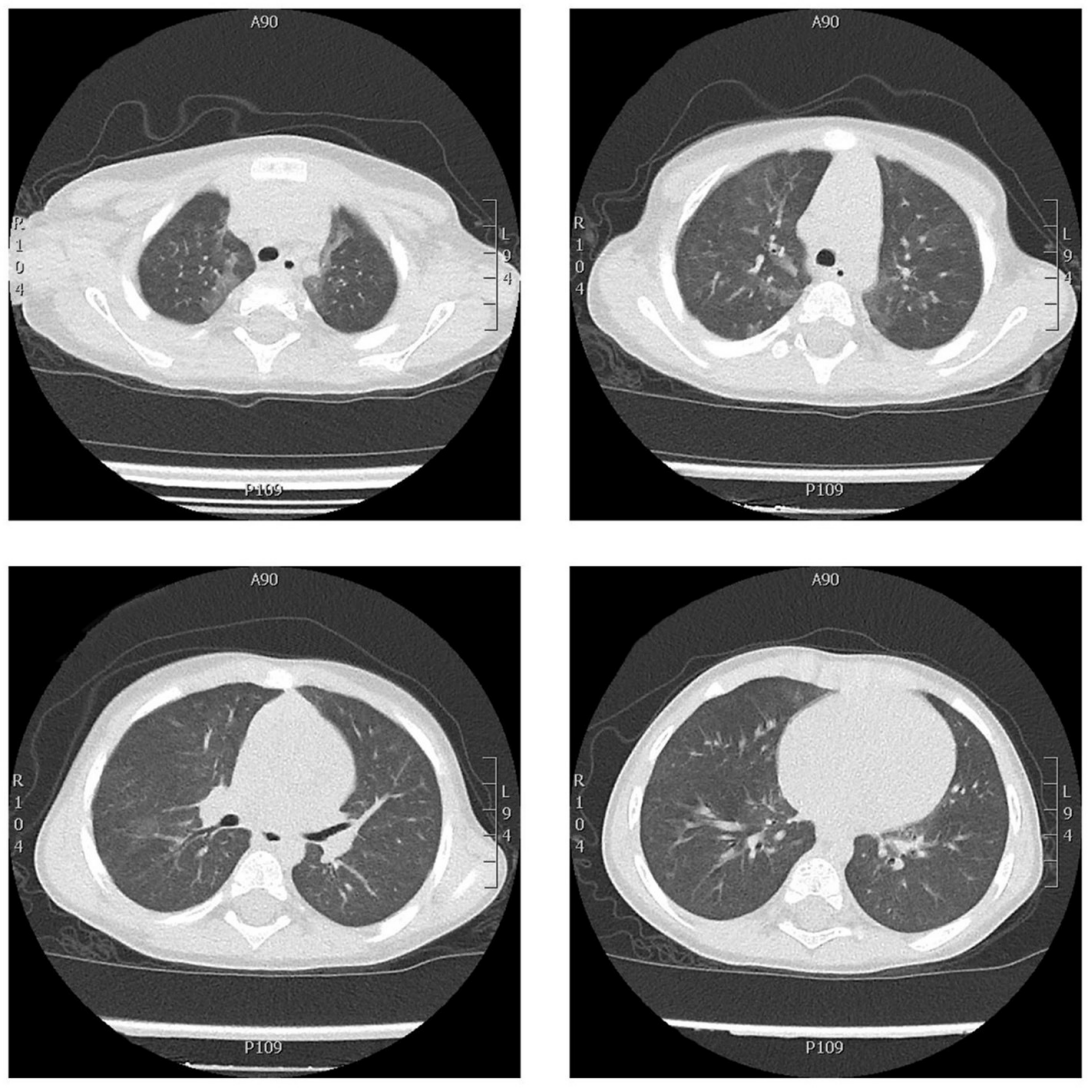
Chest CT axial scan showing ground-glass thickening in both lungs, more evident in the upper and the lower left lobes, less extended and more nuanced than the CT scan performed 6 months before. No signs of interstitial fibrosis.

He was admitted for the second time to our hospital. Serology (influenza virus A and B, and respiratory syncytial virus) was negative. Nasopharyngeal swab tested negative for COVID-19. COVID-19 neutralizing antibodies were 114 AU/ml (positive >15 AU/ml). Findings of blood and diagnostic exams performed on third hospitalization is showed in the Table 1. A chest CT axial scan showed areas of central and peripheral ground-glass opacity localized mainly in the left para-mediastinal area, superior and inferior lobes (Figure 2). He received 0.5–1 L/min of oxygen administered by nasal cannula because of respiratory distress and hypoxia mainly during sleep (SpO_2_ 88–89%). His condition gradually improved while in hospital. He was discharged with a prescription for oxygen therapy during sleep (0.5 L/min), oral steroid therapy (start prednisone 1 mg/kg/day) to be reduced very slowly and stopped after 2 weeks. In addition, he took a salbutamol inhalant, azithromycin (10 mg/kg/day for 5 days), and at home underwent monitoring of blood oxygen during sleep, heart rate, and chest movement with Getemed Vitaguard® VG 3100 monitor (Getemed, Teltow, Germany). The data were periodically downloaded onto the computer and the records were analyzed using VitaWin® software by an expert physician (LN) for assessment of central apnea (>20 s), desaturation (SpO_2_ <90%), and bradycardia or tachycardia.

An outpatient follow-up check-up (1 month later) showed persistent mild respiratory distress necessitating at-home inhalant oxygen (0.5 L/min) administered by nasal cannula; oral steroid (prednisone 0.5 mg/die reduced very slowly and stopped after 10 days), salbutamol inhalant and home cardiorespiratory monitoring with Getemed Vitaguard® VG 3100 monitor.

After 2 months of hospital discharge, the child was in good clinical condition and SpO_2_ during sleep was persistently normal (summarizing, 14 months from the start of infection).

## Discussion

To our knowledge, this is the first case of an infant presenting with high-risk BRUE and long-term pulmonary involvement from a SARS-CoV-2 infection. He had previously been well and had no history of risk for disease.

The term BRUE is applied when a child is asymptomatic on examination and a clinical evaluation yields no explanation for the event (5). Based on medical history, physical examination, and first-approach investigations of children with BRUE, associated conditions include gastroesophageal reflux, infectious diseases, respiratory diseases, and neurological diseases (7). Our patient had experienced multiple episodes of BRUE and was categorized as being at high-risk.

Several diverse conditions can co-occur with brief apneic events, such as upper and lower respiratory infections (8). In children over 2 months of age, respiratory viral infection would be expected to manifest itself with symptoms of upper respiratory to lower respiratory tract infection rather than as an isolated BRUE (6). The most frequent conditions after diagnostic evaluation of BRUE are lower respiratory tract infections (39.1%) (9).

At the age of 8 months, our patient presented clinical symptoms of transient infection. His parents reported various transient symptoms at home (apnea, bradypnea, skin pallor) between periods of well-being. The child was seen five times in the emergency department and was always found to be healthy. At the third emergency room visit, he was hospitalized, diagnosed with COVID-19 infection, then discharged in good health with a negative COVID-19 test. Finally, on his fifth emergency room visit he was again hospitalized and referred for recurrent BRUE (i.e., high-risk BRUE). The first CT scan showed features of SARS-CoV-2 pneumonia. At a follow-up examination 6 months later, his pneumonia had only mildly improved according to the second CT scan. Finally, a clinical follow-up examination showed that the respiratory symptoms had not yet resolved.

Routine immunization activities, both for primary cycles and boosters, as per National vaccine schedule recommendations, have be recommended in the era of COVID-19 pandemic unless the Referral Center reports specific contraindications (10). The measles-mumps-rubella (MMR) vaccine has been theorized as providing protection against COVID-19. MMR vaccinations may be offered for both prophylaxis and preventing the life-threatening complications of COVID-19 (11). This study suggested that higher mumps antibody levels lead to less severe or asymptomatic cases of COVID-19 infection. Babies <1 year-old are at higher risk of severe illness with COVID-19 than older children (12). In our child, scheduled MMR vaccination was administered after months of COVID-19 infection.

The national case-based surveillance system (as of May 8, 2020) collects data on confirmed childhood COVID-19 infection. Among 3,836 children, 13.8% of infections occur between ages 0–1 year-olds (13). In the United States, hospitalization was reported as being more common among infants under 1 year of age (15–62%) than in children aged 1–17 years (15–62%) (14). A multicenter study reported that hospital admission was inversely correlated with age (*p* < 0.01). Among COVID-19-infected infants under 1 year of age, 79% were hospitalized compared to 63% of 1- to 5-year-olds (15). Although rare, children under the age of 1 appear to be at higher risk of severe COVID-19 infection than older children. This is likely due to their immature immune system and smaller airways (16).

Studies have estimated that exposure to SARS-CoV-2 within the family accounts for 45–91% of pediatric cases of infection (14, 15, 17, 18). Our patient's family exposure was an important clinical indicator, also because two older family members had died during the first wave of the SARS-CoV-2 pandemic.

Symptom onset in family members frequently (77.8%) precedes symptom onset in infants after between 1 and 14 days (15). The clinical severity of infection varies from asymptomatic to critical. Children rarely develop a severe form of COVID-19 infection and usually have a mild clinical course with a good prognosis (12). Garazzino et al. reported that fever (37.5–39°C) was the most common symptom (82.1%), followed by a cough (48.8%), and rhinitis (26.8%) (15). In our patient, hyperpyrexia was an initial symptom that faded quickly. Despite general well-being, he experienced episodes of BRUE (high-risk BRUE) as a further manifestation of disease.

In children with COVID-19 pneumonia, the radiological findings were ground-glass opacity, mono or bilateral infiltrates, mesh shadows, and tiny nodules (19). Lu et al. reported that 18.1% of 171 enrolled children were <1-year-old. Of these, 78.1% developed pneumonia (18). Chen et al. reported a 1-year-old infant with severe COVID-19 who developed severe pneumonia (20). Garazzino et al. reported SARS-CoV-2-infected children <1 year-old were 39.3% (78.8% hospitalized) of 178 (1 day to 17 year-old). Those with severe complications were a pre-term newborn child and a 2-month-old infant (15). A recent review of the literature reported 40 new-borns with no persistent abnormalities (21). Whittaker et al. showed that children <1 year-old on admission were 27.5% of the total number of children hospitalized for pneumonia. Follow-up data were available in 37.1% but the outcome on long-term follow-up was not reported (22). To sum up, to our knowledge, no data are available for long-term follow-up in infants with pneumonia from COVID-19 infection, as in the case of our child.

Disease progression of lung damage occurs over 7–10 days (4). An adequate follow-up of at least 2 weeks was recommended in most instances so that the clinician can define the outcome (15). Short- and long-term outcomes are still unclear in newborns (21). The long-term effect of COVID-19 infection on lung parenchyma and pulmonary function remains unknown in the pediatric age group (23).

Recently, Needleman et al. described the case of a 25-day-old full-term infant with apnea, perioral cyanosis, nasal congestion, rhinorrhea, and a positive test for COVID-19 infection. He had a clinical history of mild encephalopathy. Three weeks after initial presentation, he was brought back to the hospital for the same reason and was ultimately completely cured (24). No data for further follow-up were given.

The main limitations of the present study are that it only deals with one clinical case report. However, to our knowledge, this report is the first to describe a long-term lung involvement from a COVID-19 infection in a very young child.

In our patient, the pulmonary complications of a COVID-19 infection were manifested in an unusual way. This is the first case of an infant presenting with high-risk BRUE as the only manifestation of long-term pulmonary involvement from a SARS-CoV-2 infection.

## Data Availability Statement

The original contributions presented in the study are included in the article/supplementary material, further inquiries can be directed to the corresponding author/s.

## Ethics Statement

Ethical approval was not provided for this study on human participants because it is a case report treated according to normal clinical practice. Written informed consent was obtained from the participant's legal guardian for the publication of this case report.

## Author Contributions

LN and MZ were responsible for the acquisition and interpretation of data, and drafting the manuscript. MA and MF provided assistance in data acquisition and critical revision of the manuscript. GP carried out a critical revision and supervision of the manuscript. VM dealt with data acquisition. All authors contributed to the article and approved the submitted version.

## Conflict of Interest

The authors declare that the research was conducted in the absence of any commercial or financial relationships that could be construed as a potential conflict of interest.
